# Respiratory Syncytial Virus (RSV)–Specific Antibodies in Pregnant Women and Subsequent Risk of RSV Hospitalization in Young Infants

**DOI:** 10.1093/infdis/jiab315

**Published:** 2021-06-15

**Authors:** Karoliina Koivisto, Tea Nieminen, Asuncion Mejias, Cristina Capella Gonzalez, Fang Ye, Sara Mertz, Mark Peeples, Octavio Ramilo, Harri Saxén

**Affiliations:** 1 Helsinki University Hospital and University of Helsinki, Children’s Hospital, Helsinki, Finland; 2 Center for Vaccines and Immunity, Abigail Wexner Research Institute at Nationwide Children’s Hospital, Columbus, Ohio, USA; 3 Department of Pediatrics, The Ohio State University College of Medicine, Columbus, Ohio,USA

**Keywords:** bronchiolitis, immune protection, infant, maternal antibodies, pre-F antibodies, respiratory syncytial virus

## Abstract

**Background:**

The fusion (F) glycoprotein of respiratory syncytial virus (RSV) represents the major neutralizing antigen, and antibodies against the pre-F conformation have the most potent neutralizing activity. This study aimed to assess the correlation between maternal antibody titers against the pre-F, post-F, and G glycoproteins and the child’s risk of developing severe RSV bronchiolitis early in infancy.

**Methods:**

We identified previously healthy term infants <3 months of age hospitalized with RSV bronchiolitis from December 2015 to March 2016. We measured IgG antibody titers to pre-F, post-F, and G proteins in maternal sera obtained at 9–12 weeks of pregnancy of these hospitalized infants’ mothers (n = 94) and compared them with serum antibody titers of control pregnant mothers (n = 130) whose children were not hospitalized.

**Results:**

All maternal samples (n = 224) had detectable pre-F antibodies. Pre-F antibody titers were significantly lower in mothers whose infants were hospitalized with RSV bronchiolitis compared with those mothers whose infants were not hospitalized (23.9 [range (or antibody titer range), 1.4–273.7] µg/L vs 30.6 [XXX, 3.4–220.0] µg/L; *P* = .0026). There were no significant differences in maternal post-F and G antibody titers between hospitalized and nonhospitalized infants.

**Conclusions:**

Our findings indicate that maternal pre-F antibodies are fundamental for providing immune protection to the infant.

## Teaser

Maternal serum immunoglobulin antibody titers directed to respiratory syncytial virus (RSV) pre-F glycoprotein were lower in infants less than 3 months of age hospitalized with RSV bronchiolitis than in maternal serum samples of age-matched control infants who were not hospitalized.

Respiratory syncytial virus (RSV) is the most important cause of bronchiolitis in childhood, leading to 3.4 million annual hospitalizations in children <5 years of age [1]. A substantial proportion of RSV-associated morbidity occurs in the first year of life, although reinfections in subsequent years are frequent [2]. Despite a number of well-defined risk factors for severe disease, the majority of infants hospitalized with RSV infection are previously healthy and born at term [3].

Knowledge gaps exist regarding the immunologic mechanisms associated with immune protection against RSV. Currently, there are no approved strategies for prevention of RSV infections in previously healthy infants [4]. Vaccine and monoclonal antibody development has advanced significantly in the past few years [5–8], based primarily on the resolution of the molecular structure of the RSV pre-F protein [9] and driven by the recognition of the global disease burden.

Higher titers of maternal RSV antibodies reduce the risk of infection with RSV and can delay the onset of severe illness in the first months of life [10–12]. Neutralizing antibody effectiveness in preventing severe RSV infection in infants was first demonstrated in the 1990s [13], and prophylaxis with the monoclonal antibody palivizumab is still currently the gold standard to protect high-risk infants [14]. Chu et al showed the effectiveness of transplacental RSV antibody transfer from mother to infant [15–17]. A recent study revealed that neutralizing maternal RSV immunoglobulin G (IgG) is already present in extremely preterm babies [18]. To our knowledge, few studies evaluated the association between maternal antibodies against different RSV glycoproteins measured early in pregnancy and subsequent protection from severe infection in infants.

The aim of this study was to assess whether there was a correlation between the titers of maternal antibodies against pre-F, post-F, or G proteins measured early in pregnancy and the infants’ risk of developing severe RSV bronchiolitis requiring hospitalization in the first 3 months of life, during 1 respiratory season.

## METHODS

### Study Design

Infants 3 months or younger hospitalized at Helsinki Children’s Hospital between December 2015 and March 2016 with RSV bronchiolitis recognized by the *International Statistical Classification of Diseases and Related Health Problems* (*ICD-10*) codes J21.0, J12.1, and/or J20.5 were identified. Helsinki Children’s Hospital is the only pediatric hospital in the area allowing us to capture all of the RSV hospitalizations during this time period. Electronic healthcare records (EHRs) were subsequently reviewed and virology diagnostic results verified. In this retrospective case-control study, severe RSV disease was defined as hospitalization for at least 3 consecutive days for RSV bronchiolitis. We also assessed other parameters of severity such as need for respiratory support and pediatric intensive care unit (ICU) admission. Premature infants (<37 gestational weeks) and children with any previously diagnosed major underlying condition documented in their EHR (ie, congenital heart disease, chronic lung disease) were excluded to make sure the duration of hospitalization was based only on RSV symptoms and severity.

**Figure 1.**
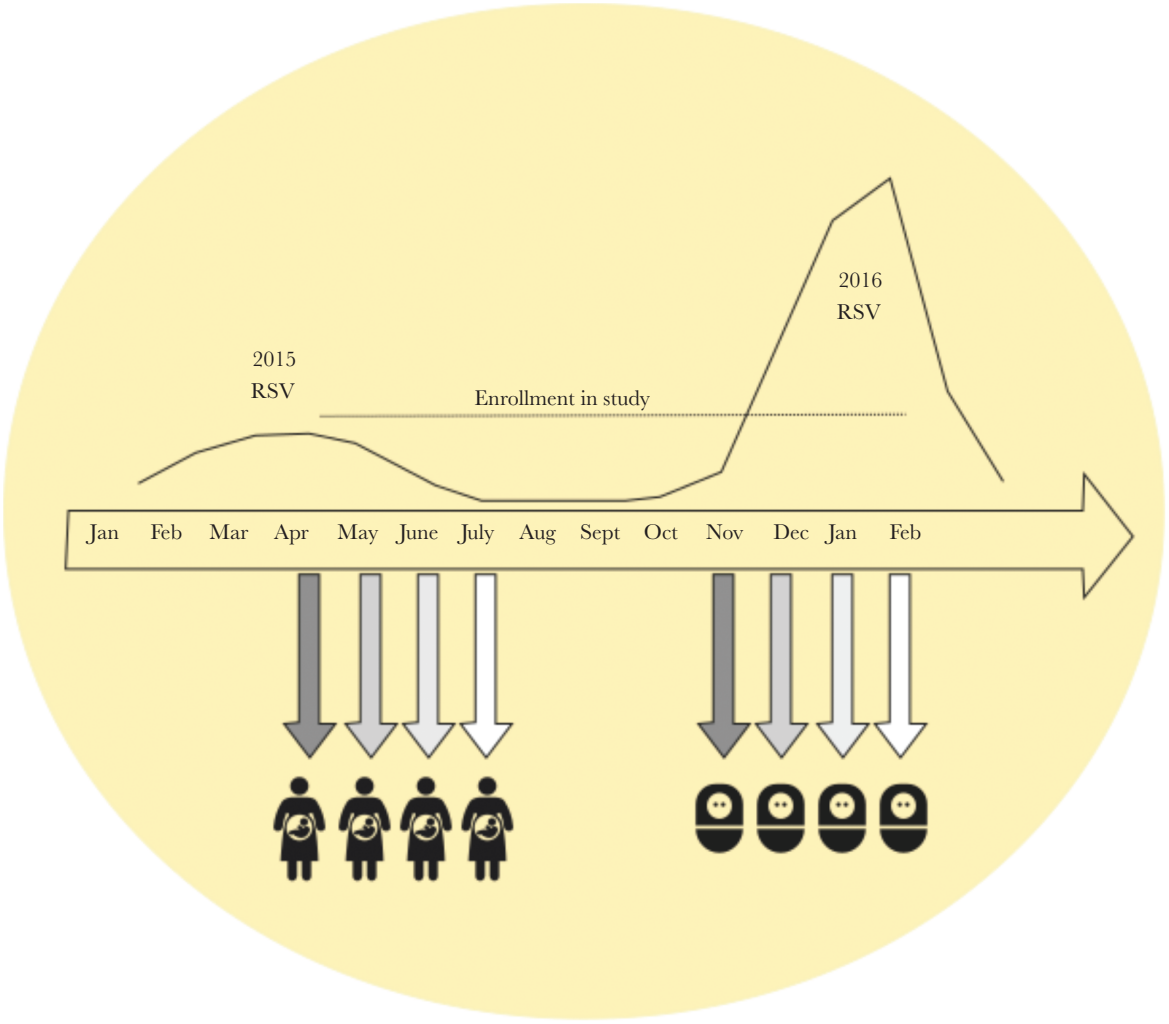
In Finland, respiratory syncytial virus (RSV) causes larger epidemics every second winter. The curve represents laboratory-proven cases of RSV infection in children aged 0–4 years during the smaller epidemic of spring 2015 and the larger epidemic the following season in 2016. The data are available in the public Infectious Disease Registry at the National Institute for Health and Welfare in Finland. The peak number of cases in March 2015 was 267 cases compared with 1192 cases in February 2016. The screening samples from the mothers were obtained at the end of the mild 2015 RSV season and the figure shows when the children of these mothers were born during the following severe 2016 RSV season.

Nearly every pregnant woman in Finland provides a serum sample in the first trimester of pregnancy (between gestational weeks 9 and 12) for screening of infectious diseases such as human immunodeficiency virus and syphilis. Usually there is a substantial volume (1–2 mL) of individual serum left over that is frozen for future research purposes. After identification of infants who met the inclusion criteria for the study, the mothers were identified from the infants’ records and the maternal screening serum samples were obtained from Helsinki University Central Hospital Laboratory for further analysis. Maternal screening serum samples obtained before April were excluded as we aimed to only include serum samples obtained after the previous RSV season (Figure 1). Serum samples from pregnant women whose infants were not subsequently hospitalized with RSV infection were selected from the screening sample bank and defined as controls (Figure 1). These control samples were obtained on the same exact day as the maternal samples of the RSV cases. At least 1 control could be found for each case, and 2 controls were selected if they were available. The EHR system in Finland enabled us to link maternal patient records to those of their children. We reviewed the records of the selected control infants thoroughly to confirm that these children were not diagnosed or hospitalized with RSV bronchiolitis. Terminated pregnancies and miscarriages were also excluded from the analysis. The screening serum samples included in the study were obtained from these pregnant women from April through July, after the mild 2014–2015 RSV season (Figure 1). It was therefore unlikely, although possible, that a mother had been infected with RSV between the time the screening sample was obtained and the time of delivery. The study protocol was approved by the Ethics Committee of the Medical Center at Helsinki University hospital.

### Laboratory Methods

All RSV infections were diagnosed with RSV rapid antigen detection tests (RADTs). The standard of care test used at Helsinki Children’s Hospital is RADT BinaxNOW RSV. RADTs have a high specificity [19].

Serum aliquots stored at –80°C were analyzed for RSV-specific antibody titers at the Abigail Wexner Research Institute at Nationwide Children’s Hospital in Columbus, Ohio. The recombinant RSV surface proteins were prepared as previously described [8]. To assure that we measure specific pre-F antibody concentrations by enzyme-linked immunosorbent assay (ELISA), we routinely use 2 pre-F–specific antibodies: a pre-F–specific monoclonal antibody (D25) and a pre-F trimer-specific antibody (AM14). An indirect ELISA assay was used to measure IgG antibody titers to pre-F, post-F, and G proteins. In brief, nickel-chelated 96-well plates were coated with 2.5 µg/mL pre-F, post-F, or G proteins, washed with phosphate-buffered saline and 0.05% Tween 20, and blocked with wash buffer containing 2% milk. Serum samples diluted in blocking buffer were added and plates were washed and incubated with 125 ng/mL goat antihuman IgG conjugated to horseradish peroxidase, followed by 3,3’,5,5’-tetramethylbenzidine. Color development was stopped with sulfuric acid, and the absorbance was quantified at 450 nm with a PE Wallac Victor 2 1420-012 microplate reader. Antibody titers in the linear range (0.5–1.5 optical density at 450 nm) were converted to µg/mL IgG from a standard curve of serially diluted human reference serum containing a known concentration of IgG (Bethyl Laboratories) assayed in parallel [20].

### Statistical Analysis

Categorical variables were compared using either the χ ^2^ test or Fisher exact test as appropriate. Continuous variables were compared using Kruskal–Wallis or Mann–Whitney tests. Results with *P* < .05 were considered significant. Statistical analyses were performed using Graph Pad Prism software version 7 (GraphPad, San Diego, California).

## RESULTS

### Pregnant Women and Infant Cohorts

We identified 227 infants aged 0–3 months hospitalized in our hospital for RSV bronchiolitis during the study period. Of these infants, 86 were excluded because the duration of hospitalization was <3 days and therefore were most likely less severe cases. In addition, 33 children were excluded because of prematurity (n = 12), a previously diagnosed neurologic disease (n = 4), congenital heart disease (n = 5), congenital anomaly (n = 5), or other underlying conditions (n = 7). A flow chart describing the exclusion process is included in Supplementary Figure 1.

Next, we located the maternal serum samples of these children’s mothers obtained during the first trimester of pregnancy. We excluded samples from mothers whose children were born in September–October (n = 12) because the maternal screening samples were obtained too early for the study design. Two maternal samples could not be located due to missing information in the patient records, leaving a total of serum samples from 94 mothers whose children were subsequently hospitalized with RSV bronchiolitis with <3 months of age (cases). Antibody titers were compared with 130 serum samples obtained from control mothers. Demographic data of the offspring are shown in Table 1.

**Table 1.** Characteristics of RSV Cases and Controls

Characteristic	RSV, No.	(%)	Controls, No.	(%)	*P* Value
Total No.	94	(100)	130	(100)	
Maternal age, y, median	30.46	…	31.77	…	.12
Month of maternal sample					.99
April	15	(16)	15	(12)	
May	26	(28)	42	(32)	
June	35	(37)	49	(38)	
July	15	(16)	19	(15)	
August	3	(3)	5	(4)	
Offspring demographic					
Male sex	50	(53)	63	(48)	.50
Birthweight, g, median	3.546	…	3.473	…	.52
Gestational age, wk					.26
37	6	(6)	7	(5)	
38	10	(11)	16	(12)	
39	31	(33)	30	(23)	
40	28	(30)	44	(34)	
41	15	(16)	25	(19)	
≥42	4	(4)	8	(6)	
Siblings (≥1)	79	(84)	67	(52)	<.0001

Abbreviation: RSV, respiratory syncytial virus.

Infants hospitalized with severe RSV infection were comparable with controls regarding gestational age, birth weight, and age of mother. There were no significant differences in serum antibody titers according to the month of the year (April, May, June, July, or August) when the maternal samples were obtained.

Children hospitalized for RSV infection were more likely than controls to have 1 or more siblings (*P* < .0001). The median age at the time of hospitalization was 35 days (interquartile range [IQR], 22.8–51.3 days). Eight of the 94 hospitalized children (9%) required ICU care. Sixty-nine (73%) required respiratory support with nasal continuous positive airway pressure or high-flow nasal cannula oxygen therapy. Otitis media was diagnosed and treated in 46 (49%) of the hospitalized children. Twenty infants (21%) received antibiotic therapy for suspected bacterial pneumonia.

### RSV-Specific Maternal Antibody Titers

**Figure 2.**
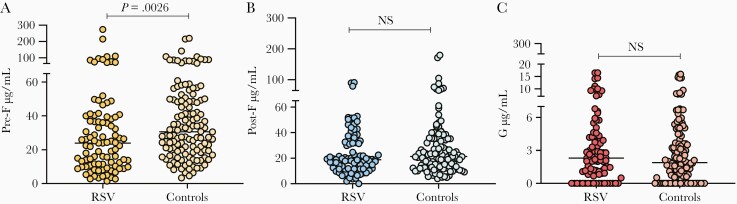
*A–C*, Respiratory syncytial virus antibody concentrations in maternal sera: cases vs controls. Abbreviations: NS, not significant; RSV, respiratory syncytial virus.

All maternal samples evaluated (n = 224) had detectable RSV antibodies against pre-F (Figure 2), with a broad range of titers across both groups. The titers of maternal pre-F antibodies were significantly lower in those mothers whose children were hospitalized with RSV bronchiolitis than in the control group (median, 23.9 [IQR, 10.6–39.3] µg/mL in the RSV group vs 30.6 [IQR, 18.9–49.3] µg/mL in the control group; *P* = .0026; Figure 2A). We performed a sensitivity analysis between the RSV group with siblings and the control group with siblings, and the overall difference in pre-F antibody titers remained in this subgroup analysis (median, 22.51 [IQR, 10.8–36.5] µg/mL in the RSV group vs 28.47 [IQR, 19.4–48.2] µg/mL in the controls; *P* = .0037). Next, we compared pre-F antibody titers in the group requiring respiratory support (n = 69) vs the controls (n = 130), and the maternal pre-F titers in the cases were significantly lower than in the controls (median, 22.44 [IQR, 9.9–39.0] µg/mL in the RSV group receiving respiratory support vs 30.58 [IQR, 18.9–49.3] µg/mL in the controls; *P* = .0016).

On the other hand, there were no significant differences in the titers of maternal post-F antibodies (median 18.8 µg/mL in the RSV group vs 21.4 µg/mL in the control group; *P* = .181) or maternal G antibodies (2.3 µg/mL in the RSV group vs 1.9 µg/mL in the control group; *P* = .372) between the 2 groups (Figure 2B and 2C). Overall, pre-F and post-F antibody titers were >10 times higher than G antibody titers (Figure 2). There were no significant differences in maternal serum antibody titers between the group needing respiratory support and the group that did not.

**Figure 3.**
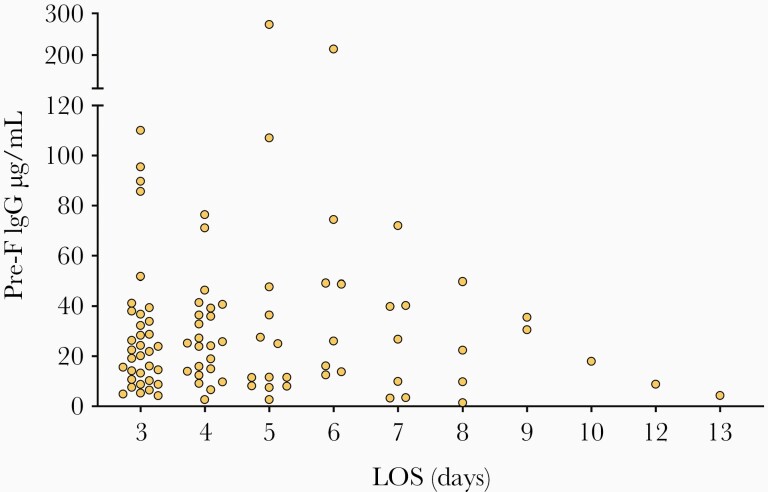
Infant hospitalization days and maternal pre-F antibodies. Abbreviations: IgG, immunoglobulin G; LOS, length of stay.

Next, we analyzed antibody concentrations according to the duration of hospitalization (length of stay), but no significant correlation was identified. A trend toward lower pre-F antibody concentrations was observed in the group requiring longer hospitalizations (Figure 3). No such trends were observed for post-F and G antibodies (data not shown).

**Figure 4.**
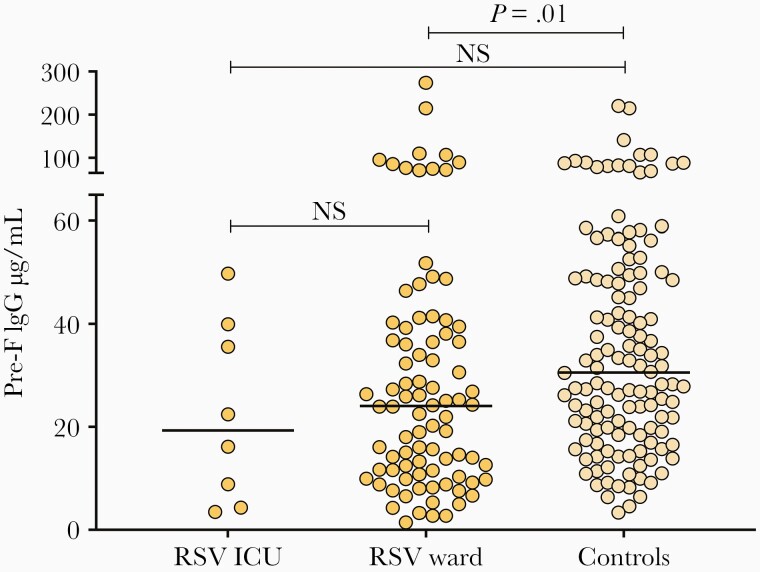
Maternal pre-F antibodies of hospitalized respiratory syncytial virus–infected infants in the intensive care unit (ICU) or ward, and maternal antibodies from the control group. Difference between groups calculated by Kruskal–Wallis test followed by Dunn test to adjust for multiple comparisons (ICU vs ward, *P* > .99; ICU vs controls, *P* = .28). Abbreviations: ICU, intensive care unit; IgG, immunoglobulin G; NS, not significant; RSV, respiratory syncytial virus.

Last, we compared the maternal antibody titers of RSV-infected children hospitalized in the ICU (n = 8) or in the inpatient ward (n = 86) and controls (n = 130). Although a trend toward lower pre-F antibody concentrations in children in the ICU group was observed (Figure 4), there were no significant differences between children admitted to the ICU and the other 2 groups.

## DISCUSSION

In the present study, we measured serum antibody titers against the major RSV surface glycoproteins in samples obtained during the first trimester of pregnancy from women whose offspring were subsequently hospitalized with severe RSV bronchiolitis. We also selected control mothers who had provided similar screening serum samples on the same exact day and whose children remained healthy and were not hospitalized with RSV bronchiolitis. In Finland, RSV causes larger epidemics biannually [21–23], and the winter season of 2015–2016 was a severe season. The health record system in Finland, combined with the unique infrastructure that biobanks blood samples collected from pregnant women, allowed us to retrospectively locate and analyze the maternal samples for specific antibody testing against RSV. We observed that lower maternal pre-F antibody titers early in pregnancy were associated with an increased risk of severe RSV in their offspring by the age of 3 months or younger. Our findings support the hypothesis that in infants, pre-F antibodies are key for providing immune protection [24, 25].

Among the RSV surface glycoproteins, both the fusion (F) and attachment (G) glycoproteins are essential for viral infectivity in vivo and are the major viral proteins capable of inducing the production of neutralizing antibodies. The G protein is highly glycosylated and the least conserved of the RSV proteins, limiting its ability to induce antibodies that are broadly neutralizing. The F protein is metastable. Transition from its pre-F to post-F form causes its fusion peptide to insert into the target cell membrane, brings the virion and cell membranes together, and causes them to fuse, thereby initiating infection. Because pre-F is the active form of the F protein, antibodies to pre-F have superior neutralizing function compared to antibodies to post-F [24, 26]. In addition, the sequence of the F protein is highly conserved among the different RSV strains, making it the main candidate for development of vaccines, monoclonal antibodies, and antiviral drugs [27, 28].

Recent studies have analyzed whether cord blood antibodies confer protection from RSV infection and found no associations. In a prospective study conducted in rural Nepal, cord blood and maternal blood were collected from 310 mother–infant pairs. Children were followed 180 days from birth, and during that time 30 infants had a symptomatic RSV infection. Higher cord blood antibody titers were not found to be protective against developing an earlier or more severe RSV disease [15]. Another case-control study from Kenya found no significant differences between children hospitalized with RSV infection and controls in mean concentrations of cord blood–neutralizing antibodies. That study also had a relatively small sample of 30 hospitalized infants [29]. In addition to the limitation of small sample size, neither of those studies measured antibodies against specific RSV surface glycoproteins. What is also possible, is that titers of RSV maternal antibody after an infant’s natural exposure are not completely protective [29].

Studies addressing the role of specific RSV antibodies in infants with acute RSV infection are limited. A small study conducted in 33 infants hospitalized with severe RSV disease found no significant associations between specific anti-RSV antibodies and disease severity [30]. On the other hand, a more recent and larger study of 65 infants with acute RSV infection showed a significant association between higher concentrations of pre-F antibody and lower disease severity, assessed by a standardized clinical disease severity score [24]. In the present study, and although we measured antibodies in maternal sera instead of infant serum, of the small group of mothers whose infants required ICU care, none had high pre-F antibody titers. This observation suggests the possibility that a certain threshold of pre-F antibodies may provide protection from the most severe forms of the disease in the very young infant. This is, however, an early hypothesis that will need confirmation in future studies.

Maternal immunization has been a safe and effective approach to prevent infections in young children resulting from pathogens causing tetanus, pertussis, and influenza. The development of new vaccines against other important pathogens of infancy is gaining support as maternal vaccination is being more widely studied and accepted [31–33]. Maternal immunization against RSV is one potential strategy to protect the neonate and infant in the first months of life [34–38]. The role of antibody transfer through breast milk is also under investigation as a means to help convey protection after birth if maternal antibody levels have been boosted [25].

Passive immunization through maternal vaccination or administration of extended-half-life monoclonal antibodies are probably the most effective ways to protect RSV-naive infants in the first months of life, as active immunization of this immunologically immature group is challenging. The clinical impact of RSV infection during pregnancy and outcomes of pregnancy with RSV infection are being investigated as maternal vaccination might also decrease the disease burden in pregnant women [39–41]. In the present study, all pregnant women evaluated had evidence of preexisting RSV antibodies, although there was significant variation in titers. The presence of antibodies in pregnant women is not surprising as reinfection with RSV is common since natural infection induces partial protection [42–44]. As failed vaccination studies have suggested, it is important that the protein(s) selected for inclusion in future RSV vaccines include epitopes that induce the most neutralizing antibodies. Antibodies against numerous antigenic epitopes of the F protein have widely varying neutralization capacity. The most potent epitopes for inducing neutralizing antibodies are conformation dependent and unique to the pre-F protein [45–47], although palivizumab targeting site II shared by the pre-F and post-F conformation still remains the only US Food and Drug Administration–approved monoclonal antibody for protection against severe RSV disease. The recent results from the Novavax phase 3 Prepare Trial, in which 4636 pregnant women were randomized in a 2:1 ratio in the third trimester to receive a particle-based vaccine vs placebo, showed that maternal immunization against RSV in the future is a feasible strategy, even if the trial did not meet its primary endpoint [34]. In that study, RSV immunization during pregnancy was associated with protection against all-cause lower respiratory tract infection, which may be an important additional benefit of maternal RSV immunization. Continuing to explore maternal immunization in addition to further developing extended-half-life monoclonal antibodies seems crucial for preventing RSV infections in the youngest of infants.

Our study has limitations. Because of the retrospective study design, we were not able to analyze other factors that may have potentially affected disease severity such as RSV genotypes or viral loads in hospitalized children. Due to limited data regarding the number and age of siblings, we could not perform multivariate analyses to control for the contribution of siblings to the risk of disease. However, a sensitivity analysis including only children with siblings found that controls had significantly higher pre-F antibody titers than RSV cases. The RSV cases were not tested for viral coinfections as this is not a standard procedure at Helsinki Children’s Hospital. In addition, due to limited blood volumes, we did not measure neutralizing antibodies, although current evidence has shown strong correlations between pre-F antibody titers and neutralization [26], suggesting that titers of pre-F antibodies represent a reliable surrogate for neutralization. Transplacental antibody transfer might have been influenced by a variety of factors that our study was not able to capture. Breastfeeding could not be reliably assessed from the EHR, but previous studies have shown that >90% of infants are breastfed in Finland at the age of 1 month [48, 49]. Levels of neutralizing maternal RSV antibodies may be associated with RSV seasonality in temperate climates and could play a role in cyclic outbreak patterns [50]. Even though we carefully took into consideration RSV seasonality in Finland, it is possible that mothers were exposed to RSV after serum samples were obtained, and the antibody titers at the end of the pregnancy could have differed from those measured in the first trimester. We tried to account for this, however, by choosing screening serum samples obtained at the end of the previous RSV season. Nevertheless, the fact that pre-F maternal antibody titers measured during the first trimester correlated with protection against severe RSV disease is remarkable and supports the use of pre-F as a vaccine antigen for maternal immunization.

In conclusion, the findings in this study support the current understanding that maternal pre-F antibodies can protect infants from severe RSV infection. Follow-up studies are needed to explore different epitope-targeting antibodies and antibody titers needed to prevent RSV disease in infants.

## Supplementary Data

Supplementary materials are available at *The Journal of Infectious Diseases* online. Supplementary materials consist of data provided by the author that are published to benefit the reader. The posted materials are not copyedited. The contents of all supplementary data are the sole responsibility of the authors. Questions or messages regarding errors should be addressed to the author.

jiab315_suppl_Supplementary_Figure_S1

jiab315_suppl_Supplementary_Materials

## Notes

***Disclaimer.*** The sponsors had no role in study design, data collection, data analysis, data interpretation, decision to publish, or preparation of the manuscript.

***Financial support.*** This work was supported by the National Institutes of Health (NIH) (grant number AI112524 to A. M., M. P., and O. R.).

***Potential conflicts of interest.*** A. M. has received research grants from the NIH and Janssen, as well as fees for participation in advisory boards from Janssen, Sanofi Merck, and Roche. O. R. has received research grants from the NIH, the Bill & Melinda Gates Foundation, and Janssen; has received fees for participation in advisory boards from Merck, MedImmune/Sanofi-Pasteur, Lilly, and Pfizer; and has received fees for lectures from Pfizer. M. P. has received research grants from the NIH, the Cystic Fibrosis Foundation, Janssen, and Pfizer, outside the submitted work. All other authors report no potential conflicts of interest.

Presented in part: 36th Annual Meeting of the European Society for Paediatric Infectious Diseases, Malmö, Sweden, 28 May–2 June 2018.
