# Supplementary figures and images for: Respiratory Syncytial Virus (RSV)–Specific Antibodies in Pregnant Women and Subsequent Risk of RSV Hospitalization in Young Infants
Source: J Infect Dis. 2021 Jun 15;225(7):1189–96. doi: 10.1093/infdis/jiab315 (PMC8974854; doi:10.1093/infdis/jiab315)

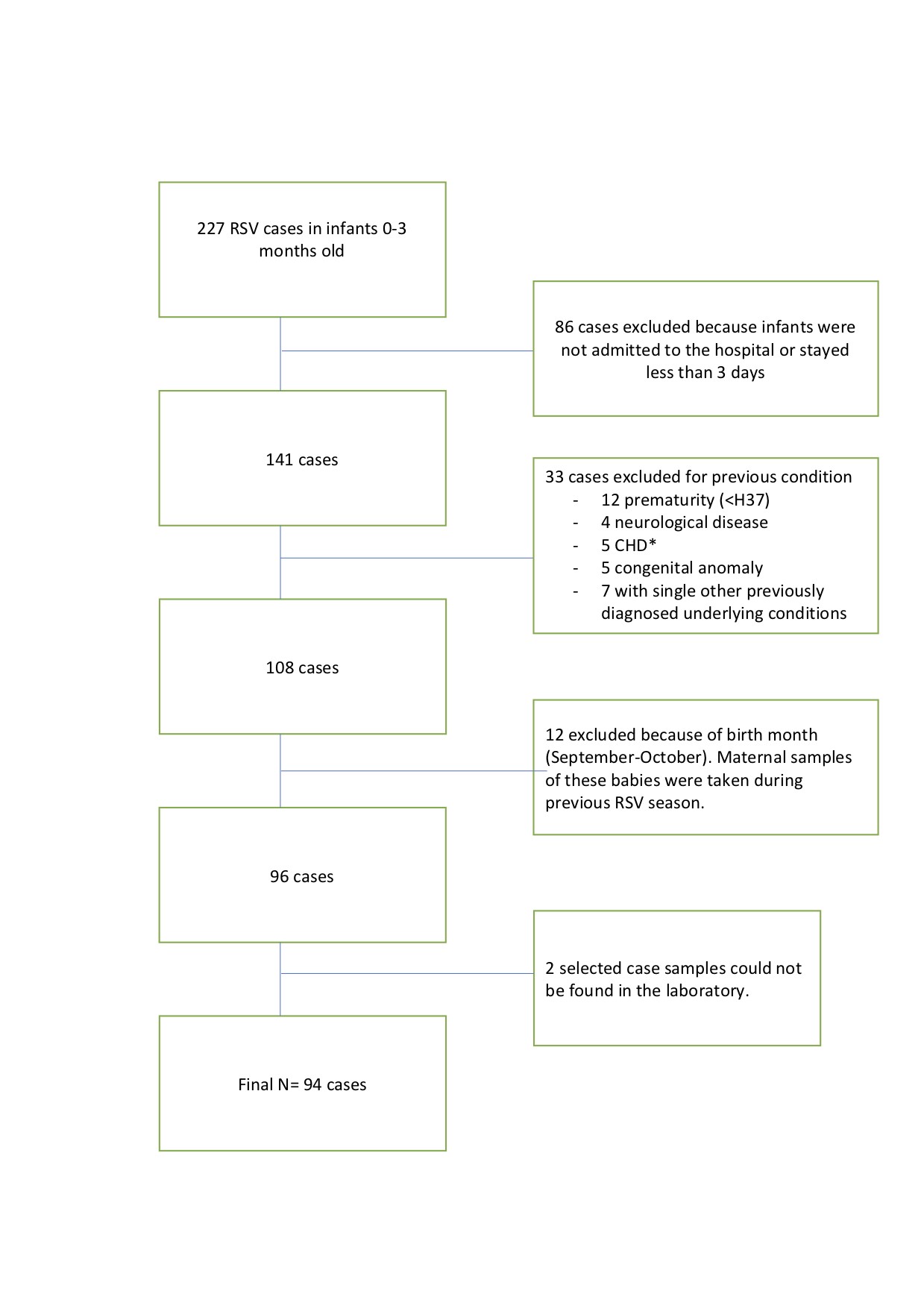

Supplement: jiab315_suppl_Supplementary_Figure_S1 [file jiab315_suppl_supplementary_figure_s1.jpeg]
