# Supplementary material for: Respiratory Syncytial Virus (RSV)–Specific Antibodies in Pregnant Women and Subsequent Risk of RSV Hospitalization in Young Infants
Source: J Infect Dis. 2021 Jun 15;225(7):1189–96. doi: 10.1093/infdis/jiab315 (PMC8974854; doi:10.1093/infdis/jiab315)
Supplement: jiab315_suppl_Supplementary_Materials [file jiab315_suppl_supplementary_materials.docx]

**Supplementary Figure 1:** 227 infants aged 0-3 months were diagnosed with RSV bronchiolitis in Helsinki Children’s Hospital between December 2015 and March 2016. After applying the exclusion criteria, 94 cases were included in the study. *CHD = Congenital Heart Disease
